# The online focus group technique for validating a theoretical model: an experience report

**DOI:** 10.1590/0034-7167-2025-0269

**Published:** 2026-06-22

**Authors:** Tatiane Cunha Florentino, Mayckel da Silva Barreto, Handerson Silva Santos, Tatiane Araújo dos Santos

**Affiliations:** I Universidade Federal da Bahia. Salvador, Bahia, Brazil; II Universidade Estadual de Maringá. Maringá, Paraná, Brazil

**Keywords:** Focus Groups, Models Theoretical, Validation Study, Methodology, Document Analysis., Grupos Focales, Modelos Teóricos, Estudio de Validación, Metodología, Análisis de Documentos

## Abstract

**Objectives:**

to report the experience of using the Online Focus Group technique in the validation of a theoretical model, built based on Grounded Theory.

**Methods:**

three focus groups were formed, whose meetings took place virtually, in a synchronous mode, from September to October 2023. The stages of the Focus Group included: 1) group composition and organization of the approach; 2) development and conduction of the groups; and 3) data analysis from the recorded media.

**Results:**

nine nurses and six nursing technicians participated. The participants’ opinions regarding the agreements and disagreements with the presented materials contributed to the reformulation of the diagram representing the theoretical model under study.

**Final Considerations:**

the Online Focus Group technique proved suitable for validating theoretical models derived from documentary research. Advantages included low cost and the possibility of participation from people in various geographical locations, as well as the recording of the meeting for later verification.

## RESUMEN

**Objetivos:**

narrar la experiencia del uso de la técnica de Grupo Focal Online para la validación de un modelo teórico basado en la Teoría Fundamentada en Datos.

**Métodos:**

se formaron tres grupos focales, cuyas reuniones se llevaron a cabo en formato virtual y modalidad sincrónica, de septiembre a octubre de 2023. Las etapas comprendieron: 1) composición del grupo y organización del enfoque; 2) desarrollo y conducción de los grupos; 3) análisis de los datos de las grabaciones.

**Resultados:**

participaron nueve enfermeros y seis técnicos de enfermería. Las opiniones de los participantes sobre las concordancias y discordancias de los materiales presentados contribuyeron a la reformulación del diagrama representativo del modelo teórico en estudio.

**Consideraciones Finales:**

la técnica del Grupo Focal Online propicia la validación de modelos teóricos derivados de la investigación documental, con ventajas en bajo costo, posibilidad de participación de personas de diversos espacios geográficos y grabación de las reuniones para comprobaciones posteriores.

## INTRODUCTION

The Focus Group (FG) can be defined as “a type of interview or conversation conducted in small, homogeneous groups with the ability to elicit opinions and values about the topic under discussion”^(1)^. The purpose of the FG is to capture participants’ perceptions, feelings, and ideas by understanding different points of view that arise from the interactional context created^(2)^. It has been used for decades in health research, especially in nursing, and therefore constitutes an important tool for data generation in qualitative investigations in the field^(3,4)^.

To ensure the effectiveness of the technique, the planning stage should prioritize the development of a guide that initially addresses the most general aspects, progressing toward more specific ones. The environment should not be directive, and the moderator should encourage the participation of all members. The composition of the group is a key factor for success and, therefore, it is recommended that it be as homogeneous as possible in terms of personal characteristics and experiences related to the phenomenon under investigation, so that discussion and interaction can be fostered^(1)^.

An FG should be conducted through a meeting with 6 to 12 participants, one moderator, and one observer. The moderator’s role is to keep the discussion focused on the topic, encourage participation, and deepen the conversation. The observer should pay attention to aspects related to group dynamics, observe non-verbal communication, and record other impressions during the discussion. Ideally, an FG should not last longer than an hour and a half^(5)^.

The FG requires interaction among participants during the meeting. Before the Covid-19 pandemic, it was predominantly conducted in person. However, a review study covering the period from 2012 to 2022 identified the use of FGs in synchronous or asynchronous written formats, as well as synchronous video/audio or audio formats^(5)^. Due to the limitations imposed by social distancing, researchers turned to an alternative to the traditional FG the Online Focus Group (OFG). Conducting OFGs poses ethical and methodological challenges, as it is not merely a transposition of the in-person environment to the virtual one. In the virtual setting, several variables can affect the achievement of FG objectives, such as internet connection quality, the participant’s physical environment (noise, interference, lack of privacy), reduced interpersonal interaction, and the risk of breaches in information confidentiality due to data leaks^(5)^.

On the other hand, the advantages of the OFG include the possibility of expanding participation due to the convenience of online access, as well as enabling the inclusion of more diverse participants-something that would be more difficult in traditional FGs, since geographical barriers are eliminated. Nevertheless, there remains a lack of empirical studies and experience reports regarding the quality of data produced through OFGs compared to traditional FGs^(4)^, as well as a scarcity of methodological reports that describe in detail how the technique has been applied for the validation of theoretical models in Nursing.

In light of the above, and considering the need for reflection on the use of digital platforms as a methodological alternative for data generation in qualitative studies-intensified by the context of social isolation during the pandemic-this study presents an experience report on the use of the OFG technique for validating a theoretical model concerning errors in the work of nursing technicians.

## OBJECTIVES

To report the experience of using the Online Focus Group technique in validating a theoretical model, built based on Grounded Theory (GT).

## METHODS

### Ethical aspects

The study was approved by the Research Ethics Committee of the Federal University of Bahia and met the requirements of Resolutions No. 466/12 and No. 510/2016 of the National Health Council, as well as the guidelines for procedures in research with any stage in a virtual environment of the National Research Ethics Commission (CONEP).

### Study design, period and setting

This is an account of an experience regarding the use of the OFG technique for validating a theoretical model on errors in the work of nursing technicians. The aforementioned theoretical model is derived from a doctoral thesis presented to the Graduate Program in Nursing and Health at the School of Nursing of the Federal University of Bahia (UFBA), linked to the Professional Error in Nursing project.

The validation of the theoretical model was carried out through the formation of three OFGs in the synchronous modality, consisting, respectively, of five nurse managers, four clinical nurses, and six nursing technicians. The recruitment of participants was based on convenience of access for the principal researcher from her network of contacts, prioritizing professionals working in public and private hospitals in Northeast Brazil, as this was the location of the main research project.

Participants were invited via messages through the WhatsApp^®^ application. Those who responded to the invitation were instructed to sign the Informed Consent Form (ICF) using the iLovePDF^®^ digital signature application. They then received the link to access the online meeting room and instructions regarding the meeting time, the need to keep cameras activated, and the importance of choosing a private location for the meeting. Additionally, they were instructed to read the Terms of Reference, which included an explanation of the analytical process, an explanation of the concept of the categories and properties that structured the theoretical model, and a diagram representing the theoretical model.

Each OFG meeting took place via the Microsoft Teams^®^ platform, from September to October 2023, in a total of three meetings with an average duration of 100 minutes each. The OFGs were fully recorded in audio and video to facilitate the analysis of the discussed content. The media derived from the OFGs were archived confidentially and kept only by the principal researcher. Both the approach and the conduct of the OFGs were carried out maintaining similar procedures, although each group had specific characteristics and a different number of participants. The experience was lived by a doctoral student and two professors with doctoral degrees, affiliated with public universities in Bahia and Paraná. During the development of the focus groups, a moderator and two observers were present. To conduct this study, the guidelines of the Consolidated criteria for reporting qualitative research (COREQ) were followed, as per the guide for studies with a qualitative approach.

The focus group was structured in three phases: 1) group composition and organization of the approach; 2) development and conduction of the focus groups; and 3) data analysis from the recorded media.

## RESULTS

### Characterization of participants

Fifteen healthcare workers participated in the focus groups, including 9 nurses and 6 nursing technicians. Of these, 12 were women and 3 were men.

Regarding their professional setting, 3 worked as nurse managers in public hospitals and 2 in private hospitals; 2 nurses worked in private hospital care and 2 in public hospitals; and 3 nursing technicians worked in public hospitals and 3 in private hospitals.

In terms of geographical distribution, the majority resided and worked in the state of Bahia (12 participants), and the others in the states of Sergipe, Ceará, and Rio Grande do Norte (one participant from each state).

During the focus group with the nurse managers, they were at their workplaces, and at one point, one of the participants needed to turn off her camera due to a work interruption, which led to the requirement that in subsequent groups, participants should not be on duty during the focus group sessions.

### Group composition and organization of the approach

In this stage, the professional categories that would participate in the validation process were defined, as well as the number of meetings and the digital platform that would facilitate the meetings.

The essential prerequisite was that they were performing work activities in hospitals, since the theoretical model developed in the doctoral study characterized the error strictly within the hospital environment. At this stage, of all the workers invited (n=23), eight did not attend the scheduled online meeting.

In order to better organize and direct the dialogue in the Focus Group, it was decided to hold three meetings, one meeting for each participant profile. In this experience, the Microsoft Teams^®^ platform was considered a neutral environment for the Focus Groups, due to its ease of access for participants and its ability to control access to the online room, as well as audio and video recording and automatic transcription of the audio, if necessary. This stage allowed for the evaluation of the online technology, analyzing the limitations and possibilities for scientific research.

For the development of the Focus Group technique, the principal researcher was defined as the first moderator, with the second moderator being one of the professors responsible for supervising the doctoral thesis, while the other professor acted as an observer. The moderators were responsible for initiating the presentation of the topic to be discussed and encouraging participation and in-depth discussion by the group. The observer was responsible for paying close attention to facial expressions and intonation of speech.

### Development and facilitation of focus groups

The focus group discussions (FGDs) began with a camera and microphone test for the participants, as it was required that they keep their cameras on to capture facial expressions, emotions, and their concentration on the topic discussed. Initially, participants were welcomed and thanked for accepting the invitation, followed by an introduction of each participant and an explanation of the roles of the moderators and observers, as well as clarifications about the FGD dynamics. Additionally, the objectives of the thesis, the methodological steps taken, and the diagram representing the theoretical model under study were presented. Following this, the following trigger question was posed: Do the categories, properties, and diagram presented represent the daily reality of services that contribute to the occurrence of errors in the work of nursing technicians? Subsequently, the group discussion began.

In the FGD composed of nurses in management positions, the participants affirmed that the categories and properties that made up the theoretical model were satisfactory, but they offered criticisms regarding the configuration of the diagram, suggesting strategies for better visual representation in accordance with the phenomenon presented, which were accepted by the researcher. The participants also suggested demonstrating in the diagram how the categories connected, replacing dotted unidirectional arrows with solid bidirectional arrows, and introducing a line that represented a dynamic cycle for the occurrence of the studied phenomenon.

The restructured diagram, based on the feedback from the first FGD, was presented to the two subsequent groups, composed of nurses and nursing technicians working in hospital care in the states of Sergipe and Bahia. There was unanimous agreement regarding the categories and properties, as well as the theoretical model.

The average duration of the FGDs was 100 minutes, and the sessions were concluded by the moderators whenever it was identified that no new information was being generated about the context represented by the explanatory diagram. In conclusion, the moderator thanked the participants, provided necessary clarifications, and explained how the group’s participation contributed to validating the phenomenon under study.

At the end of the process, it was understood that the explanatory diagram proposed in the primary research was representative of the daily work reality in the hospital environment, demonstrating the capacity of the developed research to understand and reveal the phenomenon through its results. Therefore, it was considered unnecessary to conduct further focus group discussions.

### Analysis of data from recorded media

The analysis of the recordings made by the principal researcher highlighted, as an advantage of conducting the Focus Group Discussions (FGDs), the possibility of verifying the participants’ expressions later. It was noticeable that, during the FGD discussions, they remained focused on the speeches and questions, and there was effective participation from everyone.

Throughout the first FGD, the moderators’ participation was more present because this group pointed out the necessary modifications to the diagram representing the theoretical model. This, however, did not happen during the other groups, with the intention that the discussion would remain driven by the participants. Thus, in the second and third groups, the moderators’ interventions were punctual, only when necessary to guide the discussion or for a better understanding of the discourse.

During the analysis, it was understood that the discussions and experiences expressed converged with the phenomenon represented in the theoretical model. Furthermore, they provided guidance on how some categories used in this model are expressed in the context and daily life of nursing work and how each professional category understands the organization of the nursing work process and the factors contributing to errors in the work of nursing technicians.

## DISCUSSION

The experience gained in using the online focus group (OFG) technique proved effective for validating theoretical models derived from documentary research. Although the focus group technique is mostly applied in person, the virtual environment proved useful, although adaptations in its conduct were necessary^(4)^.

Among the adaptations experienced for the OFG in this research, the need to explicitly state in the invitation to participate the choice of a quiet location with minimal interruptions for access to the virtual room stands out. The lack of control over the environment can impair participants’ attention and hinder the flow of the discussion^(6)^. This point represented a challenge for the researchers, as the participants were workers in the nursing field with different work schedules and who, in some cases, required access to the OFG room during working hours.

Although the in-person focus group has the advantage of allowing control of the environment for immersion in the discussion, it makes it impossible to bring together people with difficulty accessing the meeting in person, both due to geographical distance and social factors. Considering that in this study the participants were workers in the nursing field, participation was facilitated by the use of the virtual tool, since in the group of managers, specifically, all were at work during the group session, although one of the participants needed to turn off the camera for a few minutes, which did not harm the progress of the discussion.

Regarding the feasibility of online technology for conducting the OFG technique, other benefits included low cost, the possibility of recording the meeting, and ease of access to the virtual room. The choice of platform for conducting the meetings prioritized free access and accessibility. The use of tools familiar to the participants reduces the possibility of dropout and increases self-confidence in participating more actively in the discussions^(4)^. During the meetings, there was no difficulty accessing the platform due to its layout or problems with the internet connection. In this experience, as the object of study involved the work process of nursing technicians, the groups were divided into three virtual meetings with different participants, in order to ensure the exchange of experiences without confrontation due to the hierarchical context imposed by the very structure of the nursing work process in relation to nursing technicians. It is essential that the approach during the Focus Group Discussion (FGD) is comfortable for the group and does not result in unavoidable discussions and refusal to express any opinions. Therefore, the formation of groups with common characteristics is necessary, given that some variables may influence the results of the study^(7)^.

In this study, because it deals with a complex topic - errors in the work of nursing technicians - and because the data source that comprised the analytical process of the Grounded Theory was legal documents - Ethical Disciplinary Processes - the need arose to highlight the researcher’s methodological and interpretive capacity regarding the reality and dynamics of the phenomenon. It is worth noting that the construction of the theory is based on inductions to produce concepts from the data and deductions to generate hypotheses and understand the relationships between the concepts derived from the data, based on the researcher’s interpretation^(8,9)^.

It should be noted that the use of the FGD for validation of the theoretical model was important because, although in the Ethical Disciplinary Processes (EDP) the researchers had access to the data of the accused, complainants, and witnesses, for ethical reasons, the condition imposed by the Regional Nursing Councils for access to the EDP was not to make any contact with those involved in order to protect them.

Thus, the combination of the FGD technique was a marker for creating spaces for dialogue, identifying agreements, disagreements, and new questions for refinement and validation of the theory^(10)^. The FGD proved sufficient to achieve the objectives proposed by the studied topic, through the realization of a single meeting per participant group.

To ensure a deeper discussion and facilitate the mediation of the Focus Group (FG), the intervention adopted in this research was the development of a Terms of Reference reinforcing the invitation to participate, containing a brief summary of the study conducted and consisting of the concept of the FG and the term category. Furthermore, the Terms described the properties that characterized the explanatory categories of the studied phenomenon and the diagram proposed to guide the FG discussion. It is considered that making the subject to be discussed in the FG explicit guides and empowers participants to reflect beforehand and, consequently, enables broad participation during the dialogue, in addition to allowing them to decide whether or not to participate in the group^(4)^.

It is also possible to point out that conducting the FG in three stages contributed to the mediators’ maturation in using the technique, allowing for greater skill in incorporating more complex questions based on the elaborations made by the participants during the discussion at each meeting. Additionally, it encouraged them to feel free to express their opinions with minimal interference.

Thus, reflecting on the experience with the FG, it is emphasized that the use of the technique provided the opportunity to improve the illustration of the diagram representing the theoretical model, since, although the participants of the first FG showed agreement regarding the categories involved in the phenomenon, illustrative adjustments were suggested to better represent the dynamics surrounding the researched topic, with the diagram subsequently validated in the subsequent FGs. The compatibility of the FG technique with the validation stage of the Grounded Theory in studies whose data source is documentary is also highlighted. Regarding the groups’ agreement with the presented theoretical model, the researchers’ capacity for abstraction and anchoring to the data is considered.

### Study limitations

Although the research has generated knowledge, increasing the participation of workers from other Brazilian states could contribute to a deeper understanding of the topic.

### Contributions to the fields of nursing, health or public policy

This study contributed to presenting and reflecting on the Focus Group Interview (FGI) as an effective and efficient technique for conducting qualitative research in nursing, involving participants in different geographical areas.

## FINAL CONSIDERATIONS

The online focus group technique (OFG) proved suitable for validating the theoretical model derived from studies using Grounded Theory.

The OFG allowed participants to express agreements and disagreements about the categories and the diagram, which contributed to the reformulation of the diagram representing the theoretical model after the first focus group and validation in subsequent groups. Furthermore, it made it possible to collectively reflect on how the categories represented in the diagram manifested themselves in the participants’ work context, providing a space for the construction and expression of lived experiences in the world of work, based on the central category and the categories shown in the diagram.

The use of the OFG technique demands adequate planning, specifically for the validation of theoretical models. It is relevant to present the topic to the participants in advance to guide the discussion. Another point of attention is the need for participants to have time dedicated exclusively to online participation and in a private and quiet location to reduce interruptions and maintain everyone’s concentration. Researchers must ensure preparation and knowledge about the technique to allow the group to feel empowered to express opinions without judgment during the session.

There is a need to expand the use of OFG as a technique for validating theoretical models in other studies, including participants from other Brazilian states, in order to deepen knowledge on the subject.

## Data Availability

The research data are not available.
