# Supplementary material for: The online focus group technique for validating a theoretical model: an experience report
Source: Rev Bras Enferm. 2026 Jun 22;79:e20250269. doi: 10.1590/0034-7167-2025-0269 (PMC13317006; doi:10.1590/0034-7167-2025-0269)
Supplement: Translation

# Translation: A técnica de grupo focal *on-line* para validação de modelo teórico: relato de experiência

**DOI:** 10.1590/0034-7167-2025-0269pt

**Authors:** Tatiane Cunha Florentino, Mayckel da Silva Barreto, Handerson Silva Santos, Tatiane Araújo dos Santos

**Affiliations:** I Universidade Federal da Bahia. Salvador, Bahia, Brasil; II Universidade Estadual de Maringá. Maringá, Paraná, Brasil

**Keywords:** Grupos Focais, Modelos Teóricos, Estudo de Validação, Metodologia, Análise Documental.

## Abstract

**Objetivos:**

relatar a experiência do uso da técnica de Grupo Focal *On-line* na validação de um modelo teórico, construído com base na Teoria Fundamentada nos Dados.

**Métodos:**

foram conformados três grupos focais, cujos encontros aconteceram virtualmente, em modalidade síncrona, de setembro a outubro de 2023. As etapas do Grupo Focal compreenderam: 1) composição do grupo e organização da abordagem; 2) desenvolvimento e condução dos grupos; e 3) análise dos dados a partir das mídias gravadas.

**Resultados:**

participaram nove enfermeiros(as) e seis técnicos(as) em enfermagem. A manifestação dos participantes acerca das concordâncias e discordâncias dos materiais apresentados contribuiu para reformulação do diagrama representativo do modelo teórico em estudo.

**Considerações Finais:**

a técnica do Grupo Focal *On-line* demonstrou-se adequada para validação de modelos teóricos oriundos de pesquisa documental. Destacaram-se, como vantagens, o baixo custo e a possibilidade de participação de pessoas de variados espaços geográficos, bem como de gravação da reunião para verificações posteriores.

## INTRODUÇÃO

O Grupo Focal (GF) pode ser conceituado como “um tipo de entrevista ou conversa em grupos pequenos e homogêneos com capacidade para extrair a opinião e valores sobre o tema discutido”^(1)^. O objetivo do GF é captar percepções, sentimentos e ideias dos participantes, a partir da compreensão de pontos de vista distintos, advindos do próprio contexto de interação criado^(2)^. Tem sido utilizado nas pesquisas da área da saúde, em especial da enfermagem, há décadas e, por isso, constitui importante ferramenta para a produção de dados em investigações qualitativas na área^(3,4)^.

Para garantir a efetividade da técnica, a etapa do planejamento deve priorizar a elaboração de um roteiro que busque considerar inicialmente seu aspecto mais geral, avançando em direção ao mais específico. O ambiente não deve ser diretivo, e o moderador deve estimular a participação de todos. A formação do grupo é uma etapa determinante para o êxito e, para tanto, recomenda-se ser o mais homogêneo possível em termos de características pessoais e vivências no fenômeno em investigação, a fim de que o debate das experiências e a interação possam ser fomentados^(1)^.

Um GF deve ser operacionalizado por meio de uma reunião, com 06 a 12 participantes, com um moderador e um observador. O moderador tem papel de manter o foco no tema, promover a participação de todos e aprofundar a discussão. O observador deve atentar-se para aspectos relacionados à condução do grupo, observar as comunicações não verbais e relatar outras impressões durante o debate. O ideal é que a duração de um GF não ultrapasse uma hora e meia^(5)^.

O GF exige interação entre os participantes em uma reunião. Antes da pandemia da Covid-19, era realizado, predominantemente, de modo presencial. Contudo, estudo de revisão com recorte temporal de 2012 a 2022 identificou o uso do GF nas modalidades síncrono ou assíncrono por escrito; síncrono por vídeo/áudio ou áudio^(5)^. Com as limitações impostas pelo distanciamento social, pesquisadores recorreram à modalidade alternativa ao GF tradicional, ou seja, ao Grupo Focal *On-line* (GFO). A realização do GFO apresenta desafios éticos e metodológicos, pois não se trata apenas de uma transposição do ambiente presencial para o virtual. No ambiente virtual, algumas variáveis podem interferir no alcance dos objetivos do GF, como qualidade da conexão da internet, espaço onde o participante estará (ruídos, interferências, ausência de sigilo), possibilidade de menor interação entre as pessoas e risco de perda do sigilo das informações no ambiente virtual, por vazamento de dados^(5)^.

Em contrapartida, as vantagens do GFO incluem a possibilidade de ampliar a participação, pela comodidade de acessar à reunião *on-line*, bem como de permitir a inclusão de participantes mais diversos, o que seria uma dificuldade no GF tradicional, visto que a barreira geográfica é rompida. Contudo, ainda há carência de pesquisas empíricas e relatos de experiência sobre a qualidade dos dados produzidos pelo GFO em detrimento do GF tradicional^(4)^, além de escassez de relatos metodológicos que descrevam com profundidade como tem sido aplicado para validação de modelos teóricos em Enfermagem.

Diante do exposto, e partindo da necessidade da reflexão sobre o uso de plataformas digitais como alternativa metodológica na produção de dados em estudos qualitativos, intensificada pelo contexto de isolamento social decorrente da pandemia, apresenta-se o presente estudo, caracterizado com um relato de experiência a respeito da técnica do GFO para validação de modelo teórico sobre o erro no trabalho de técnicos(as) em enfermagem.

## OBJETIVOS

Relatar a experiência do uso da técnica de Grupo Focal *On-line* na validação de um modelo teórico, construído com base na Teoria Fundamentada nos Dados (TFD).

## MÉTODOS

### Aspectos éticos

O estudo foi aprovado pelo Comitê de Ética e Pesquisa da Universidade Federal da Bahia e atendeu aos requisitos das Resoluções nº. 466/12 e nº. 510/2016 do Conselho Nacional de Saúde, bem como às orientações para procedimentos em pesquisas com qualquer etapa em ambiente virtual da Comissão Nacional de Ética em Pesquisa (CONEP).

### Desenho, período e local do estudo

Trata-se de um relato de experiência a respeito da utilização da técnica GFO para validação de um modelo teórico sobre erro no trabalho de técnicos(as) em enfermagem. O referido modelo teórico é derivado de uma tese de doutorado apresentada ao Programa de Pós-Graduação em Enfermagem e Saúde da Escola de Enfermagem da Universidade Federal da Bahia (UFBA), vinculada ao projeto Erro Profissional em Enfermagem.

A validação do modelo teórico foi realizada mediante a formação de três GFO na modalidade síncrona, constituídos, respectivamente, por cinco enfermeiros(as) gestores, quatro enfermeiros(as) assistenciais e seis técnicos(as) em enfermagem. O recrutamento dos participantes foi por conveniência de acesso da pesquisadora principal a partir de sua rede de contatos, prezando por profissionais com vínculo de trabalho em hospitais públicos e privados do Nordeste do Brasil, por se tratar do lócus da pesquisa matriz.

Enviou-se convite aos participantes por mensagens pelo aplicativo *WhatsApp* ^®^. Aqueles que retornaram ao convite foram instruídos a assinar ao Termo de Consentimento Livre e Esclarecido (TCLE) mediante assinatura digital do aplicativo *iLovePDF* ^®^. Em seguida, receberam o *link* para acesso à sala *on-line* e orientações sobre o horário da reunião, necessidade de manutenção das câmeras ativadas e de escolha por local reservado para este encontro. Adicionalmente, foram instruídos sobre a leitura do Termo de referência, composto pela explicação do processo analítico, explanação sobre o conceito das categorias e propriedades que estruturaram o modelo teórico e diagrama representativo do modelo teórico.

A reunião de cada GFO aconteceu via plataforma *Microsoft Teams* ^®^, no período de setembro a outubro de 2023, em um total de três encontros com duração média de 100 minutos cada. Os GFO foram integralmente gravados por áudio e vídeo, a fim de facilitar a análise do conteúdo discutido. As mídias derivadas dos GFO foram arquivadas em sigilo e mantidas em posse apenas da pesquisadora principal. Tanto a abordagem como a condução dos GFO foram realizadas mantendo procedimentos similares, embora cada grupo apresentasse características específicas e número de participantes diferentes.

A experiência foi vivenciada por uma estudante de doutorado e dois professores(as) doutores(as), vinculados(as) a universidades públicas na Bahia e no Paraná. Durante o desenvolvimento dos GFO, estavam presentes um moderador e dois observadores. Para condução deste estudo, seguiu-se o preconizado pelo *Consolidated criteria for report qualitative research* (COREQ), conforme o guia para estudos com abordagem qualitativa.

O GFO foi estruturado em três fases: 1) composição do grupo e organização da abordagem; 2) desenvolvimento e condução dos grupos focais; e 3) análise dos dados a partir das mídias gravadas.

## RESULTADOS

### Caracterização dos participantes

Participaram dos grupos focais 15 trabalhadores(as), sendo 9 enfermeiros(as) e 6 técnicos(as) de enfermagem. Dentre esses, 12 eram mulheres e 3 homens.

Com relação ao local de atuação profissional, 3 atuavam como enfermeiros(as) gestores de hospitais públicos e 2 de hospitais privados; 2 enfermeiros(as) atuavam na assistência hospitalar privada e 2 em hospitais públicos; e 3 técnicos(as) em enfermagem trabalhavam em hospitais públicos e 3 em hospitais privados.

Quanto à distribuição geográfica, a maioria residia e atuava no estado da Bahia (12 participantes), e os demais nos estados de Sergipe, Ceará e Rio Grande do Norte (um participante de cada estado).

Durante o GFO dos enfermeiros(as) gestores, eles estavam em seus locais de trabalho e, em um momento, uma das participantes precisou fechar a câmera por interrupção no trabalho, o que levou à exigência que nos grupos posteriores os participantes não estivessem em período de jornada laboral.

### Composição do grupo e organização da abordagem

Nessa etapa foram definidas as categorias profissionais que participariam do processo de validação, o número de encontros e a plataforma digital que viabilizaria as reuniões.

A prerrogativa essencial era que estivessem desenvolvendo atividades laborais em hospitais, já que o modelo teórico elaborado no estudo de doutorado caracterizava o erro estritamente no ambiente hospitalar. Nesta etapa, de todos os trabalhadores convidados (n=23), oito não compareceram no momento agendado para a reunião *on-line*.

Com vistas a melhor organizar e direcionar o diálogo no GFO, definiu-se a realização de três reuniões, contemplando uma reunião por perfil de participantes. Nessa experiência, considerou-se como ambiente neutro para os GFO a plataforma *Microsoft Teams* ^®^, pela facilidade de acesso por parte dos participantes e por permitir o controle de acesso à sala *on-line,* além da gravação de áudio, vídeo e transcrição automática dos áudios, caso necessário. Esta etapa possibilitou a avaliação da tecnologia *on-line*, analisando as limitações e possibilidades para a pesquisa científica.

Para o desenvolvimento da técnica de GFO, foi definido o pesquisador principal como primeiro moderador, sendo o segundo moderador representado por um dos professores responsáveis pela orientação da tese de doutorado, ao passo que o outro professor ocupou o lugar de observador. Os moderadores foram responsáveis por iniciar a apresentação do tema a ser discutido e encorajar a participação e o aprofundamento da discussão pelo grupo. Ao observador, coube o olhar atento às expressões faciais e entonação das falas.

### Desenvolvimento e condução dos grupos focais

Os GFO tiveram início com o teste de câmera e microfone dos participantes, pois havia a exigência de permanecer com a câmera ligada para capturar expressões faciais, emoções e a concentração dos participantes ao tema discutido. Inicialmente, desejou-se boas-vindas e foram feitos agradecimentos aos participantes pelo aceite ao convite, seguidos da apresentação de cada um deles e da explicação da função dos moderadores, observadores, além de esclarecimentos sobre a dinâmica do GFO. Adicionalmente, foram apresentados os objetivos da tese, as etapas metodológicas realizadas e o diagrama representativo do modelo teórico em estudo. A partir disto, realizava-se a seguinte pergunta disparadora: as categorias, propriedades e o diagrama apresentado representam o cotidiano dos serviços que favorecem a ocorrência do erro no trabalho de técnicos(as) em enfermagem? Na sequência, iniciava-se o diálogo grupal.

No GFO constituído por enfermeiros(as) alocados(as) em cargos de gestão, os participantes afirmaram que as categorias e propriedades que conformavam o modelo teórico estavam satisfatórias, mas apresentaram críticas acerca da configuração do diagrama, sugerindo estratégias para melhor representação visual em concordância com o fenômeno apresentado, as quais foram acatadas pela pesquisadora. As participantes sugeriram ainda a demonstração no diagrama de como as categorias se conectavam, a substituição de setas unidirecionais pontilhadas por setas bidirecionais sólidas, além da introdução de uma linha que representasse um ciclo dinâmico para ocorrência do fenômeno estudado.

Foi apresentado aos dois grupos subsequentes, compostos por enfermeiros(as) e técnicos(as) em enfermagem com ocupação na assistência hospitalar nos estados de Sergipe e Bahia, o diagrama reestruturado com base nos apontamentos do primeiro GFO. Houve concordância unânime acerca das categorias e propriedades, assim como do modelo teórico.

O tempo médio de duração dos GFO foi de 100 minutos, e os encerramentos eram conduzidos pelos moderadores sempre que identificado esgotamento de novas informações sobre o contexto representado pelo diagrama explicativo. Ao finalizar, o moderador agradecia aos participantes, fazia esclarecimentos necessários e acerca de como a participação do grupo conduzia a validação do fenômeno estudado.

Ao final do processo, entendeu-se que o diagrama explicativo proposto na pesquisa primária era representativo da realidade cotidiana de trabalho no ambiente hospitalar, demonstrando capacidade da pesquisa desenvolvida em compreender e revelar o fenômeno por meio de seus resultados. Dessa forma, considerou-se desnecessária a realização de outros GFO.

### Análise dos dados a partir das mídias gravadas

A análise das gravações realizadas pela pesquisadora principal evidenciou, como vantagem da realização do GFO, a possibilidade da verificação das expressões dos participantes posteriormente. Foi notório que, durante a discussão dos GFO, eles mantiveram-se concentrados às falas e questionamentos e houve a participação efetiva de todos.

Ao longo do primeiro GFO, a participação dos moderadores se fez mais presente em virtude deste grupo ter apontado as modificações necessárias ao diagrama representativo do modelo teórico. Este fato, contudo, não aconteceu durante os demais grupos com a intenção de que a discussão permanecesse sustentada a partir dos participantes. Dessa forma, no segundo e terceiro grupos, as intervenções dos moderadores foram pontuais, apenas quando necessário direcionar a discussão ou para melhor entendimento do discurso.

Durante a análise, apreendeu-se que as discussões e vivências expressadas convergiam com o fenômeno representado no modelo teórico. Ademais, forneciam direcionamentos da forma como algumas categorias utilizadas neste modelo se expressam no contexto e cotidiano do trabalho em enfermagem e de como cada categoria profissional compreende a organização do processo de trabalho em enfermagem e os fatores contribuintes para o erro no trabalho de técnicos(as) em enfermagem.

## DISCUSSÃO

A experiência vivenciada na utilização da técnica do GFO demonstrou-se efetiva para validação de modelos teóricos oriundos de pesquisa documental. Embora a técnica do GF seja aplicada majoritariamente de forma presencial, o ambiente virtual confirmou-se útil, ainda que sejam necessárias adaptações na sua condução^(4)^.

Dentre as adaptações vivenciadas para o GFO nesta pesquisa, destaca-se a necessidade de explicitar, no convite para participação, a escolha por um local silencioso e com o mínimo de interrupções para acesso à sala virtual. A ausência de controle do ambiente pode prejudicar a atenção dos participantes e favorecer o desaquecimento da discussão^(6)^. Este ponto significou um desafio para os pesquisadores, pois os participantes eram trabalhadores(as) do campo da enfermagem com diferentes jornadas de trabalho e que, em alguns casos, exigiram o acesso à sala do GFO durante o horário de trabalho.

Embora o GF presencial apresente vantagem por permitir o controle do ambiente para imersão na discussão, impossibilita a reunião de pessoas com dificuldade de acesso presencial, tanto pela distância geográfica como por fatores sociais. Considerando que neste estudo os participantes eram trabalhadores do campo da enfermagem, a adesão foi facilitada pelo uso da ferramenta virtual, uma vez que no grupo de gestores, especificamente, todos encontravam-se em período laboral durante a sessão grupal, embora um dos participantes tenha apresentado a necessidade de fechar a câmera por alguns minutos, o que não representou prejuízo para o andamento da discussão.

Ainda sobre a viabilidade da tecnologia *on-line* para realização da técnica de GFO, outros benefícios incluíram o baixo custo, a possibilidade de gravação da reunião e a facilidade de acesso à sala virtual. A escolha da plataforma para o desenvolvimento das reuniões priorizou a gratuidade e acessibilidade. A utilização de ferramentas familiares aos participantes reduz a possibilidade de desistência e aumenta a autoconfiança em participar, de maneira mais ativa, das discussões^(4)^. Durante os encontros, não houve dificuldade de acesso à plataforma pelo seu *layout* tampouco por problemas na conexão de internet.

Nessa experiência, como o objeto de estudo envolvia o processo de trabalho de técnicos(as) em enfermagem, os grupos foram divididos em três encontros virtuais com participantes distintos, a fim de assegurar a troca de experiências sem a confrontação pelo contexto hierárquico imposta pela própria conformação do processo de trabalho em enfermagem em relação aos técnicos(as) em enfermagem. Torna-se imprescindível que a abordagem durante o GFO seja confortável para o grupo e não resulte em discussões incontornáveis e com recusa a qualquer emissão de opiniões. Dessa forma, a constituição de grupos com características em comum é necessária, dado que algumas variáveis podem influenciar os resultados do estudo^(7)^.

Neste estudo, por se tratar de um tema complexo - erro no trabalho de técnicos(as) em enfermagem - e pelo fato da fonte de dados que compôs o processo analítico da TFD ter sido documentos legais - Processos Éticos Disciplinares -, surgiu a necessidade de evidenciar a capacidade metodológica e de interpretação do pesquisador sobre a realidade e a dinâmica do fenômeno. Vale destacar que a construção da teoria se fundamenta em induções para produzir conceitos a partir dos dados e deduções para gerar hipóteses e compreender as relações entre os conceitos derivados dos dados, a partir da interpretação do pesquisador^(8,9)^.

Cabe informar que o uso do GFO para validação do modelo teórico se fez importante, pois, embora nos Processos Éticos Disciplinares (PED) os pesquisadores obtivessem acesso aos dados das denunciadas(os), denunciantes e testemunhas, por questões éticas, o condicionante dos Conselhos Regionais de Enfermagem para acesso aos PED era o de não realizarem de qualquer contato com os envolvidos para preservação dos mesmos.

Dessa forma, a combinação da técnica de GFO foi um marcador para criação de espaços de diálogo, levantamento de concordâncias, discordâncias e novos questionamentos para refinamento e validação da teoria^(10)^. O GFO demonstrou-se suficiente para alcançar os objetivos propostos pelo tema estudado, a partir da realização de um único encontro por grupo de participante.

Para garantir o aprofundamento da discussão e viabilizar a mediação do GFO, foi adotada como intervenção nesta pesquisa a elaboração de um Termo de Referência reforçando o convite à participação, contendo breve resumo do estudo realizado e constituído pelo conceito de GF e do termo categoria. Ademais, o Termo descrevia as propriedades que caracterizavam as categorias explicativas do fenômeno estudado e do diagrama proposto para guiar a discussão do GFO. Considera-se que deixar explícito o assunto a ser tratado no GFO conduz e instrumentaliza os participantes à reflexão previamente e, por conseguinte, viabiliza ampla participação durante o diálogo, além de deliberar sobre a decisão em participar ou não do grupo^(4)^.

É possível apontar ainda que a realização do GFO em três momentos contribuiu para o amadurecimento dos mediadores com a utilização da técnica, permitindo, a cada encontro, maior habilidade em incorporar questionamentos mais complexos mediante as elaborações realizadas pelos participantes durante a discussão. Adicionalmente, favoreceu que se sentissem livres para expor as opiniões com a mínima interferência.

Assim, ao refletir sobre a experiência com o GFO, enfatiza-se que o uso da técnica oportunizou o aprimoramento da ilustração do diagrama representativo do modelo teórico, pois, embora os participantes do primeiro GFO apresentassem concordância quanto às categorias envolvidas no fenômeno, foram sugeridos ajustes ilustrativos para melhor representar a dinâmica que envolve o tema pesquisado, sendo o diagrama, posteriormente, validado nos GFO subsequentes. Ressalta-se ainda a compatibilidade da técnica do GFO com a etapa de validação da TFD em estudos cuja fonte de dados é documental. Acerca da concordância dos grupos com o modelo teórico apresentado, pondera-se a capacidade de abstração e ancoragem aos dados dos pesquisadores.

### Limitações do estudo

Embora a pesquisa tenha gerado conhecimento, ampliar a participação de trabalhadores de outros estados brasileiros pode favorecer o aprofundamento da temática.

### Contribuições para as áreas da enfermagem, saúde ou políticas públicas

Este estudo contribuiu para apresentar e refletir sobre o GFO enquanto técnica eficaz e efetiva para a realização de pesquisas qualitativas em enfermagem, envolvendo participantes em diferentes espaços geográficos.

## CONSIDERAÇÕES FINAIS

Considera-se que a técnica de GFO *on-line* mostrou-se adequada para a validação do modelo teórico oriundo de estudos que utilizam a Teoria Fundamenta nos Dados.

A realização do GFO permitiu que os participantes manifestassem concordâncias e discordâncias acerca das categorias e do diagrama, fato que contribuiu para reformulação do diagrama representativo do modelo teórico após o primeiro Grupo Focal e validação nos grupos subsequentes. Além disso, tornou possível refletir coletivamente sobre como as categorias representadas no diagrama se revelavam no contexto de trabalho dos participantes, sendo um espaço de construção e manifestações das experiências vividas no mundo do trabalho a partir da provocação da categoria central e das categorias demonstradas no diagrama.

A utilização da técnica do GFO demanda adequado planejamento, especificamente, para validação de modelos teóricos, sendo relevante a apresentação antecipada do tema aos participantes, a fim de direcioná-los à discussão. Outro ponto de atenção é a necessidade de que os participantes estejam com tempo dedicado exclusivamente à participação *on-line* e em local reservado e silencioso, a fim de reduzir interrupções e manter a concentração de todos. Cabe aos pesquisadores assegurar preparação e conhecimento sobre a técnica para permitir que, durante a condução, o grupo sinta-se autorizado a manifestar opiniões sem julgamentos.

Há necessidade de expandir a realização de GFO como técnica para validação de modelos teóricos em outros estudos, incluindo participantes de outros estados brasileiros, com o propósito de aprofundar o conhecimento sobre a temática.

## Data Availability

Os dados de pesquisa não estão disponíveis.
